# Can the geriatric nutritional risk index predict the risk of osteoporosis in the elderly? A systematic review and meta-analysis

**DOI:** 10.12669/pjms.41.4.11539

**Published:** 2025-04

**Authors:** Wanghao Liu, Xiaoying Sun

**Affiliations:** 1 Wanghao Liu, Department of Endocrinology, Huzhou Third Municipal Hospital, the Affiliated Hospital of Huzhou University, 2088 Tiaoxi East Road, Huzhou, Zhejiang Province 313000, P.R. China; 2 Xiaoying Sun, Department of Endocrinology, Huzhou Third Municipal Hospital, the Affiliated Hospital of Huzhou University, 2088 Tiaoxi East Road, Huzhou, Zhejiang Province 313000, P.R. China

**Keywords:** Elderly, Malnutrition, Nutritional status, Osteoporosis

## Abstract

**Objective:**

The elderly are at a high risk of malnutrition as well as osteoporosis. In this review, we examined if the geriatric nutritional risk index (GNRI) can predict the risk of osteoporosis in the elderly.

**Methods:**

In this PRISMA compliant systematic review we searched the electronic databases of PubMed, Embase, ScienceDirect, and Web of Science. The search included studies from inception of databases to December 29, 2023. All studies examining the association between GNRI and osteoporosis were included.

**Results:**

Seven studies were selected. All studies were cross-sectional in design. Meta-analysis of all seven studies showed that low GNRI was significantly associated with the risk of osteoporosis in elderly patients (OR: 1.33 95% CI: 1.15, 1.53). The interstudy heterogeneity was high as the I^2^ value was 87%. Results did not change on sensitivity analysis. Subgroup analysis based on study location, presence of diabetes, sample size, GNRI cut-off, method to determine cut-off, and diagnostic criteria for osteoporosis showed mixed results.

**Conclusion:**

Low GNRI can be a simple marker for predicting osteoporosis in the elderly. Current evidence is from a few studies with a high risk of bias.

## INTRODUCTION

Osteoporosis is a commonly diagnosed metabolic bone disease. About 1.2 billion people in the world suffer from osteoporosis.^1^ The disease is associated with reduced bone mass and microstructure alterations which make bones susceptible to fractures.^2^ Data from China shows that about 19% of the elderly suffer from osteoporotic fractures and the prevalence has significantly increased in the past decade.^3^ With increasing lifespan and higher prevalence of osteoporosis, the number of fragility fractures is going to increase in the coming years. Importantly, osteoporotic fractures are associated with significant morbidity and mortality.^4^

Malnutrition is also highly prevalent amongst the elderly.^5^ Further, it has a significant impact on the health, cognitive and physical functioning, and quality of life of the elderly.^6^ Several cohort studies have shown that malnourished individuals have an increased risk of osteoporosis.^7^ȓ^9^ However, it is unclear which malnutrition assessment tool can best predict such risk. One of the commonly used geriatric nutritional assessment tools is the Geriatric Nutritional Risk Index (GNRI). The GNRI was developed in 2005 and it combines albumin and body weight data to assess disease prognosis.^10^ The advantage of GNRI is that is derived from easily available measurements and can provide an accurate and rapid assessment of the nutritional status of the elderly.^11^ GNRI has been shown to aid in prognostication of patients with respiratory, renal, and cardiac diseases.^12^ȓ^15^ In incident hemodialysis patients, GNRI was found to independently predict cardiovascular mortality.^12^ The marker has also been associated with all-cause and cardiac mortality in coronary artery disease patients undergoing percutaneous coronary intervention.^13^ A meta-analysis also shows that GNRI predicts mortality and cardiovascular events in heart failure patients.^14^ It also has good prognostic ability in predicting outcomes in cancer patients.^16^ȓ^18^ In this context, GNRI has also been used to predict the risk of osteoporosis in the elderly by several studies.^11^,^19^,^20^ However, the results have been incoherent and there is no consensus on its use to predict osteoporosis. The current study pooled evidence from the literature to provide high-quality evidence on the predictive ability of GNRI for osteoporosis in the elderly.

## METHODS

A literature search was conducted by two reviewers from inception of databases to December 29, 2023. The databases used were PubMed, Embase, ScienceDirect, and Web of Science. The reference lists of the included studies were also explored.

The review protocol was registered on PROSPERO (CRD42023495457) and is based on the Preferred Reporting Items for Systematic Reviews and Meta-Analyses (PRISMA) reporting guidelines.^21^ We included only human studies which:

- Examined the risk of osteoporosis in the cohort based on GNRI.
- Reported the association as odds/risk/hazard ratio with 95% confidence intervals (CI) or reported data to calculate the same.
- Osteoporosis was diagnosed based on bone scans. 4) published in the English language. Studies not specific to GNRI were excluded. Studies not on osteoporosis, reporting only T scores, and not providing complete data were also excluded.

Keywords used were “Geriatric nutritional risk index”, “T score”, “osteoporosis”, “osteopenia”, AND “bone mineral density”. The search query generated for all databases was: “(T score) OR (bone mineral density) OR (osteopenia) OR (osteoporosis)) AND (Geriatric nutritional risk index)”. First, we removed duplicate results from the search. Then the reviewers read the titles and abstracts of remaining studies for initial selection. Studies relevant to the review were identified. These underwent full-text analysis for final selection. Discrepancies were discussed and resolved.

### Data and study quality:

We removed the following information from studies: author name, year, country, study type, type of patients included, sample size, mean age, male gender, GNRI cut-off, method to determine the cut-off, diagnostic criteria for osteoporosis, percentage with osteoporosis, factors adjusted in the analysis of outcome, follow-up (if a cohort study), and effect size. Studies were examined for risk of bias using the Newcastle Ottawa Scale (NOS).^22^ Studies were judged for representativeness of the study cohort, comparability of groups, and measurement of outcomes.

### Statistical analysis:

The meta-analysis was done on “Review Manager” (RevMan, version 5.3). We calculated odds ratio (OR) and 95% confidence intervals (CI) of the association between GNRI and osteoporosis. A sensitivity analysis involving the removal of one study at a time was conduxted. Publication bias was checked with funnel plots. The I^2^ statistics determined inter-study heterogeneity. I^2^ >50% indicated substantial heterogeneity. Subgroup analysis was done based on study location, presence of diabetes, sample size, GNRI cut-off, method to determine cut-off, and diagnostic criteria for osteoporosis.

## RESULTS

**Fig.1:**
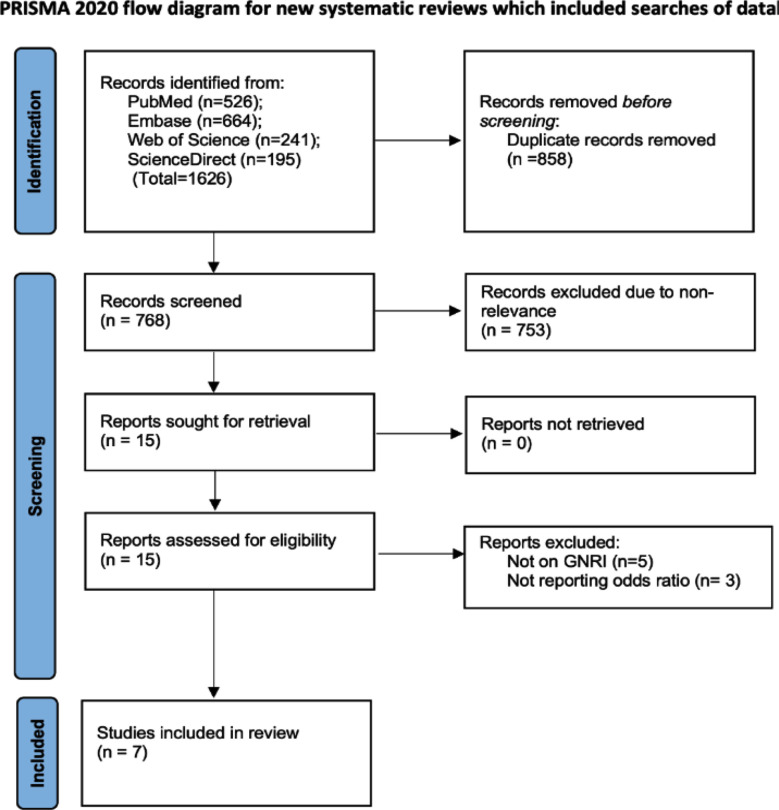
PRISMA flowchart of the study showing steps of selection

We found 1626 articles after the final search (Fig.1). Finally, seven studies^11^,^19^,^20^,^23^–^26^ fulfilled the inclusion criteria. Most of the included studies were from China, the others were from the USA, Korea, and Japan (Table-I). All studies were cross-sectional in design. Also, all studies included elderly patients with mean age ranging from 60.5 to 71.6 years. Four studies included only patients with diabetes mellitus (DM), one study included only hemodialysis patients, one study included only rheumatoid arthritis patients and one study included all elderly patients. Four studies used the GNRI cut-off of 98 to define malnourished patients. The remaining studies used values of 95, 96, or 99.5. Six studies used pre-determined values to define the cut-off while one study used the receiver operating characteristic curve to obtain the cut-off. Most studies used the 1994 World Health Organization (WHO) criteria^27^ to diagnose osteoporosis. Most studies received a score of seven on the NOS scale. One study received a score of five.

**Table-I:** Details extracted from included studies.

Study	Location	Type	Included patients	Sample size	Male gender (%)	Age (years)	GNRI cut-off	Determination of cut-off	Diagnosis of OP	Percentage with OP	Adjusted factors	NOS score
Sun 2023^11^	China	CS	Elderly patients with DM	525	19.2	71.6	98	Standard value	1994 WHO criteria	67.2	Sex, smoking, drinking, age, BMI, albumin, creatinine, uric acid, blood glucose, triglycerides, high density lipoprotein,	7
Liang 2023^23^	China	CS	Elderly patients with DM	290	51.4	71	98	Standard value	Guidelines for the Use of Bone Metabolic Markers in the Diagnosis and Treatment of Osteoporosis (2012 edition)	37.4	Course of diabetes, Vitamin D, glycated hemoglobin, serum S-CTX	7
Ji 2022^20^	China	CS	Elderly patients with DM	610	NR	66	98	Standard value	1994 WHO criteria	25.9	Gender, age, the duration of diabetes, fasting plasma glucose, Vitamin D, Procollagen of type-1 N-propeptide, and albumin	7
Huang 2022^19^	USA	CS	Elderly patients	7405	51.9	70	98	Standard value	1994 WHO criteria	8.5	Age, sex, race, education level, marital status, BMI, smoker, alcohol user, glucocorticoid user, physical activity, blood calcium, hypertension, cancer, diabetes	7
Wang 2020^26^	China	CS	Elderly patients with DM	447	50.3	66.1	99.5	ROC curve	1994 WHO criteria	15.1	Age, diabetes duration, cholesterol, triglyceride, lipoprotein, creatinine, uric, glycosylated hemoglobin, fasting blood glucose	7
Lee 2020^25^	Korea	CS	Elderly patients on hemodialysis	131	54.2	66.2	96	Standard value	1994 WHO criteria	38.9	None	5
Tokumoto 2018^24^	Japan	CS	Rheumatoid arthritis patients	146	17.1	60.5	95	Standard value	The Japanese Society for Bone and Mineral Research definition	38.4	Age, gender, disease duration, glomerular filtration rate, does of steroids, Disease Activity Score-28-CRP, Simplified Disease Activity Index, Modified Health Assessment Questionnaire, C-reactive protein	7

CS, cross-sectional; GNRI, geriatric nutritional risk index; BMI, body mass index, OP, osteoporosis; NOS, Newcastle Ottawa scale, ROC, receiver operating characteristic, WHO, world health organization; DM, diabetes mellitus.

**Fig.2:**
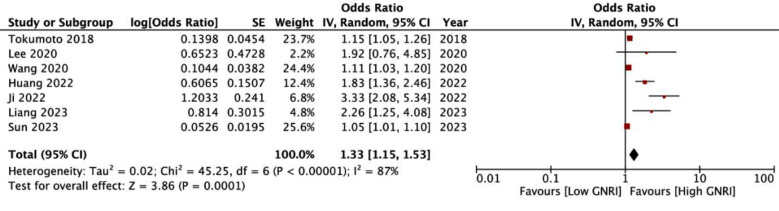
Meta-analysis forest plot demonstrating an association between low GNRI and risk of osteoporosis.

**Supplementary Fig.1:**
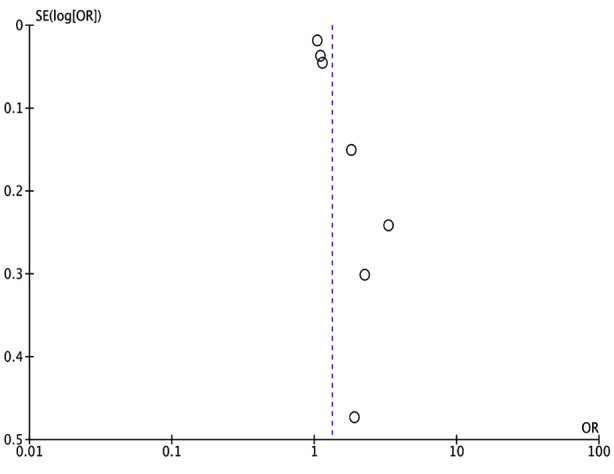
Funnel plot to examine publication bias.

Meta-analysis of all seven studies showed that low GNRI was significantly associated with the risk of osteoporosis in elderly patients (OR: 1.33 95% CI: 1.15, 1.53 I^2^=87%) (Fig.2). The funnel plot did not show publication bias (Supplementary Fig.1). Results of the sensitivity analyses are shown in Table-II. The effect size was found to range from 1.19 to 1.55 and the results remained statistically significant. The outcomes of the subgroup analysis are shown in Supplementary Table-I. Based on location, the results of Chinese studies were found to be significant but not of non-Chinese studies. Similar results were noted in subgroup analysis based on diabetes. On subgroup analysis based on sample size, results were significant for smaller sample-sized studies (<500) but not for larger studies. Also, results were non-significant when a GNRI cut-off of <98 was used but remained significant for a cut-off of ≥98. Results did not change in significance based on the method of determination of cut-off. On subgroup analysis based on diagnostic criteria of osteoporosis, the results were significant when 1994 WHO criteria were used but not for other studies.

**Table-II:** Sensitivity analysis.

Excluded study	Odds ratio [95% Confidence intervals]
Sun 2023(11)	1.55 [1.24, 1.93]
Liang 2023(23)	1.28 [1.11, 1.47]
Ji 2022(20)	1.19 [1.07, 1.33]
Huang 2022(19)	1.24 [1.08, 1.42]
Wang 2020(26)	1.51 [1.22, 1.88]
Lee 2020(25)	1.32 [1.14, 1.52]
Tokumoto 2018(24)	1.46 [1.20, 1.77]

**Supplementary Table-I:** Subgroup analysis.

Variable	Groups	Studies	Odds ratio [95% Confidence intervals]	I^2^ (%)
Location	Chinese Non-Chinese	4 3	1.32 [1.08, 1.60] 1.48 [0.99, 2.22]	90 80
Diabetes	With DM Without DM	4 3	1.32 [1.08, 1.60] 1.48 [0.99, 2.22]	90 80
Sample size	>500 <500	3 4	1.80 [0.96, 3.35] 1.17 [1.04, 1.32]	94 85
GNRI cut-off	≥98 <98	6 2	1.44 [1.18, 1.75] 1.20 [0.91, 1.57]	91 14
Method of cut-off	Standard value ROC	7 1	1.51 [1.22, 1.88] 1.11 [1.03, 1.20]	89 -
Diagnosis of osteoporosis	WHO criteria Other	6 2	1.38 [1.14, 1.68] 1.51 [0.79, 2.88]	89 80

WHO, world health organization; DM, diabetes mellitus; GNRI, geriatric nutritional risk index; ROC, receiver operating characteristic,

## DISCUSSION

The results of this systematic review indicate that low GNRI is associated with significantly higher risk of osteoporosis in elderly. The results seem robust on sensitivity analysis. However, the results were variable on subgroup analysis based on different confounders. The elderly are often at a high risk of malnutrition. Advanced age, concomitant illnesses, use of multiple medications, reduced mobility and cognition, psychological stress, and reduced appetite are some of the risk factors.^28^ However, malnutrition is often not distinguishable from age-related changes and the majority of the patients remain undiagnosed.^29^ Malnutrition has also been linked with the development of osteoporosis in narrative reviews.^30^ Malnourished patients also have a significantly higher risk of mortality as compared to normally nourished osteoporotic patients.^31^ Given the risk, malnourished elderly individuals must be identified early so that appropriate interventions can be undertaken to reduce the risk of osteoporosis and subsequent fractures.^32^

In our review, we examined the role of GNRI in predicting the risk of osteoporosis. Meta-analysis demonstrated that low GNRI was significantly associated with higher risk of osteoporosis. The results had high heterogeneity but were found to be stable during sensitivity analysis indicating a strong association between the two. The high inter-study heterogeneity is concerning and could be related to the baseline variations in the included studies. The inclusion criteria, diagnostic criteria of osteoporosis, and the cut-off for GNRI were not coherent among the studies. On subgroup analysis based on several factors, we noted that the results turned non-significant for several variables. The effect size was non-significant for non-Chinese studies, studies not on DM, GNRI <98, and sample size >500. Nevertheless, the lower end of the 95% CI was still close to one for all these subgroups indicating that the results may be non-significant due to the reduced number of studies. The overall number of studies was just seven and the subgroups may not have adequate statistical power to demonstrate a significant association.

The association between GNRI and low bone mineral density (BMD) has also been recognized by other studies in the literature. Qing et al.^33^ have shown that elderly with high GNRI (i.e. normally nourished) had significantly greater total hip and lumbar spine T-scores compared to those with low GNRI. Chen et al.^34^ have also demonstrated that high GNRI was related to high BMD and T-score in a cohort of patients on hemodialysis. Chiu et al. 35 studied 50 postmenopausal women treated with thyroidectomy and found that low GNRI was linked with low lumbar spine BMD and T-score. Thus, there seems to be evidence that low GNRI denoting malnutrition can be a predictor of osteoporosis in the elderly. However, there is no clarity on what cut-off of GNRI can best predict osteoporosis. In the current review, the most common cut-off used was 98. A similar cut-off has been used by most other studies in literature and represents the standard value to determine malnourishment.^36^ Nevertheless, the number 98 is not sacrosanct and several different cut-offs have also been used in the literature depending on the cohort characteristics and derivation of cut-off based on receiver operating curve analysis.^36^ With the current data, it is not possible to determine the sensitivity and specificity of low GNRI (<98) in predicting the risk of osteoporosis and there is a need for further robust studies.

The association between GNRI and osteoporosis can be explained by utilizing two important markers of malnutrition in the tool, namely, albumin and BMI. Albumin reflects the total body protein; reduced albumin can lead to insulin-like growth factor-1 (IGF-1) reduction. IGF-1 along with growth hormone is an important regulator of bone metabolism. Growth hormone promotes the formation of young pre-chondrocyte colonies which mature in the presence of IGF-1. IGF-1 also contributes to longitudinal bone growth and cortical bone formation and helps maintain bone mass in adult life.^37^ Animal studies have shown that IGF-1 gene disruption in mice leads to a decrease in BMD, periosteal circumference, and cortical thickness. Secondly, a high intake of protein leads to increased absorption of calcium from the intestines. The role of calcium supplementation in managing osteoporosis is well studied.^38^ Lastly, BMI is a known predictive factor for osteoporosis.^39^,^40^

### Strengths of study:

The strength of the review is that it is the first to examine the role of GNRI in predicting osteoporosis in the elderly. A detailed systematic search combined with a thorough analysis has generated important evidence on the role of GNRI. Our results have important clinical significance. GNRI is an easy to calculate, readily available and inexpensive marker which can be of clinical importance in the prediction of osteoporosis in the elderly. It can therefore be used for primary screening of elderly individuals and help in early diagnosis. However, there is a need for further research to improve the robustness of the results given the limited evidence in literature. Also, future studies should be prospective and strive to derive an optimal cut-off of GNRI for predicting osteoporosis.

### Limitations of study:

Nevertheless, there are limitations. The cross-sectional nature of the data does not establish causality between malnutrition and osteoporosis. Small number of studies is also concerning. GNRI measurements were recorded only once and it is unclear how change in GNRI affects the risk. Also, the inclusion criteria were different in the studies with most including DM patients while others included patients with kidney failure and rheumatoid arthritis. Lastly, the majority of data was from China and this limits the generalizability of the results. Further cohort studies are needed to improve evidence.

## CONCLUSIONS

Low GNRI may be a predictor of osteoporosis in the elderly. Current evidence is from a few studies with a high risk of bias, hence results must be interpreted with caution.

### Authors’ contributions:

**WL:** Study design, literature search and manuscript writing.

**WL and XS:** Data collection, data analysis and interpretation, Critical Review.

**WL:** Manuscript revision and validation, Critical Review.

All authors have read, approved the final manuscript and are responsible for the integrity of the study.
